# A New Year’s Message 2023

**DOI:** 10.3390/polym15030759

**Published:** 2023-02-02

**Authors:** Shin-ichi Yusa

**Affiliations:** Department of Applied Chemistry, Graduate School of Engineering, University of Hyogo, 2167 Shosha, Himeji 671-2280, Japan; yusa@eng.u-hyogo.ac.jp

We wish you all happiness, health and progress in the new year. How much research progress did we make in 2022? Unfortunately, the effects of the pandemic did not disappear in 2022. International travel was particularly difficult. However, these problems are being gradually resolved. We hope that your research in 2023 will be successful. This Editorial deals with the most cited papers published in the 2022 in the section “Polymer Chemistry” in the journal *Polymers*.

Liu et al. [1] reported that a combination of hydrophilic polyvinylpyrrolidone (PVP) and hydrophobic poly(3-hydroxybutyric acid-*co*-3-hydroxyvaleric acid) (PHBV) was used to prepare coaxial fibers, which have the properties of optimizing the release of poorly water-soluble drugs, such as curcumin (Cur). Monolithic F1 fibers and coaxial F2 fibers were successfully prepared. During in vivo drug release, F1 fibers only needed 4 h to release Cur completely due to the water-soluble PVP, and core–shell F2 fibers could prolong the Cur release time to 24 h. This long-term dosing method improves the therapeutic utilization efficiency of poorly water-soluble Cur. Liu et al. [2] reported that the single-fluid blending electrospinning process was combined with the casting film method to fabricate a medicated double-layer hybrid to provide a dual-phase drug-controlled release profile, with ibuprofen as a common model of a poorly water-soluble drug and ethyl cellulose and PVP as the polymeric excipients. The two-layer films can be loaded with different kinds of drugs for a combination therapy. Abbas et al. [3] reported that the attenuation coefficients of the prepared silicone rubber (SR) samples showed a clear superiority in lower energy levels over other energies, and the SR’s nano-Bi_2_O_3_ was better than the corresponding SR’s micro-Bi_2_O_3_ at all discussed energies for the shielding materials. Husna et al. [4] reported a surface-modification strategy for a metal–organic framework (MOF) through grafting of a polymer with intrinsic microporosity onto the surface of UiO-66-NH_2_. The mixed-matrix membrane (MMM) with 1 wt% loading of PIM-grafted-MOF membranes showed the best CO_2_ separation performance. Nguyen et al. [5] reported the use of specific fillers for the metakaolin-based lightweight geopolymers, emphasizing the above-mentioned physical properties, and also investigated the electromagnetic shielding ability of the carbon grid built into the lightweight geopolymer structure. The most suitable materials to be used as fillers are polystyrenes, along with hollow ceramic microsphere and Liapor. Zhao et al. [6] reported that thermochromic microcapsules were prepared by in situ polymerization, and the effect of the thermochromic microcapsule content of the coatings on the properties of the paint film was investigated. This study provides a basis for the application of thermochromic coatings. Iglesias-Mejuto et al. [7] reported that reinforced alginate–hydroxyapatite (HA) aerogel scaffolds for bone tissue engineering (BTE) applications were obtained by a dual strategy that combines extrusion-based 3D printing and supercritical CO_2_ gel drying with an extra crosslinking step. Peñas et al. [8] reviewed the current strategies to modulate the thermo-mechanical, barrier and biodegradation properties of poly(butylene succinate) (PBS). Enzymatic degradation constitutes one of the most promising routes to biodegrade this polymer. Beniak et al. [9] reported the design of an experiment, the process of measuring the resistance of samples printed via a fused deposition modeling (FDM) device and measuring the maximum tensile strength of samples. Jia et al. [10] reported an efficient method to obtain low-temperature curing phthalonitrile resins with high thermal and thermo-oxidative resistance, which would be potentially useful for the preparation of high-performance cyanide resin-based composites. Chen et al. [11] reported that iodine-immobilized UiO-66-NH_2_ metal–organic framework (MOF) (UiO66@I2) nanoparticles were added to the PCL matrix under ultrasonic vibration and evaporated the solvent to obtain a polymer membrane. MOF nanoparticles could retain most of the iodine during the sample preparation and storage, while there was very little iodine left in the free iodine/poly(ε-caprolactone) (PCL) composites. Ribas-Massonis et al. [12] reviewed UV curing, which generally consists of the formation of cross-linking covalent bonds between a resin and monomers via a photoinitiated free radical polymerization reaction, obtaining a three-dimensional polymer network. One of its many applications is in the refinish coatings market, where putties, primers and clear coats can be cured faster and more efficiently than with traditional curing. Al-Ghamdi et al. [13] reported that a hybrid mesoporous magnetic chitosan nanocomposite was successfully functionalized by cysteine to prepare a cysteine (Cys) sorbent for the sorption of uranyl ions selectively and efficiently from aqueous solution and radioactive effluents. The sorbent was efficiently tested for selective uranium sorption from multicomponent acidic simulated nuclear solution. Tao et al. [14] reported that self-healing microcapsules were prepared by using melamine–formaldehyde (MF) resin as the wall material and shellac as the core material repairing agent. The hydrophilic lipophilic balance (HLB) value of the emulsifier was the most important influencing factor. Leventis reviewed polyurea aerogels, which have been demonstrated for applications in thermal and acoustic superinsulation, ballistic protection, blast-wave mitigation, and oil-spill cleanup [15]. Polyurea-crosslinked oxide aerogels are the point of departure for ceramic and metallic aerogels, while recently developed polyurea-crosslinked alginate aerogels are prime candidates for application in the decontamination of natural waters from heavy metals, such as Pb, U and so on. Liu et al. [16] reviewed the types of light conversion agents and their preparation methods, summarized the applications for light conversion films in plants and predicted the future development directions for light conversion agents and light conversion films. Antony Samy et al. [17] reported the effect of ambient temperature on the in-built residual stresses and warpage of amorphous acrylonitrile-butadiene-styrene and semi-crystalline polypropylene (PP) polymers. The enhanced warpage in PP with an increase in ambient temperature, despite the reduction in residual stress, was ascribed to crystallization and shrinkage. Lestido-Cardama et al. [18] reported an investigation of several polyester coatings intended for food contact. Firstly, Fourier-transform infrared spectroscopy with an attenuated total reflectance spectrometer, confocal Raman microscopy and liquid chromatography coupled to ion trap mass spectrometry (LC-MS^n^) were used to identify the type of coating. Fang et al. [19] reported that L-lactide was first used as raw material to dissolve thermoplastic polyurethane (TPU) under heating conditions, and Polylactic acid (PLA)-TPU copolymer (PTC) was prepared by in situ ring-opening polymerization, named PTC. The impact strength of the PLA/TPU blend was significantly improved when the synthesized PTC was used as the compatibilizer of PLA/TPU blends, compared with the PLA/TPU blend without PTC. Noè et al. [20] reported that biobased UV-curable hydrogels were developed using modified chitosan and gelatin and applied as adsorbents for the removal of As(V) and Pb(II) from an aqueous solution.

In 2022, the following Special Issues in the section “Polymer Chemistry” were successful: “Polymer Reaction Modeling and Kinetics” edited by Enrique Saldivar-Guerra and Dagmar R. D’hooge (9 published papers), “Cross-linked Polymers” edited by Łukasz Klapiszewski and Beata Podkościelna (11 published papers), “Controlled Polymerization” edited by Pavel Ivchenko (10 published papers). Further, the following Topical Collection in the section “Polymer Chemistry” was successful: “Polymeric Coatings” edited by Ilker S. Bayer (64 published papers) and “Design and Synthesis of Polymers” edited by Shin-ichi Yusa (37 published papers). These Topical Collections are now open. A number of Special Issues in the section “Polymer Chemistry” are also currently calling for papers. We look forward to your contributions.

We appreciate all authors, reviewers and readers for the journal *Polymers*. We look forward to working with you in 2023.
